# Characteristics of dysphagia among different lesion sites of stroke: A retrospective study

**DOI:** 10.3389/fnins.2022.944688

**Published:** 2022-08-24

**Authors:** Jia Qiao, Zhi-min Wu, Qiu-ping Ye, Meng Dai, Yong Dai, Zi-tong He, Zu-lin Dou

**Affiliations:** ^1^Department of Rehabilitation Medicine, The Third Affiliated Hospital of Sun Yat-sen University, Guangzhou, China; ^2^Department of Neurosurgery, The Third Affiliated Hospital of Sun Yat-sen University, Guangzhou, China; ^3^Clinical Medical College of Acupuncture, Guangzhou University of Chinese Medicine, Guangzhou, China

**Keywords:** dysphagia, stroke, different lesion sites, videofluoroscopic swallowing study, retrospective study

## Abstract

**Objective:**

This study aims to compare the characteristics of dysphagia among different lesion sites and explore the possible risk factors that are relevant to penetration and aspiration after stroke.

**Materials and methods:**

Data on patients with post-stroke dysphagia were collected. Major measures of the videofluoroscopic swallowing study included pharyngeal transit duration (PTD), pharyngeal response duration (PRD), soft palate elevation duration (SED), stage transition duration (STD), hyoid bone anterior-horizontal displacement (HAD), hyoid bone superior-horizontal displacement (HSD), upper esophageal sphincter opening (UESO), Pharyngeal Residual Grade (PRG), and Penetration Aspiration Scale (PAS). Included patients were divided into supratentorial (deep or lobar intracerebral) and infratentorial stroke groups. The Kruskal–Wallis test, Spearman’s correlation analysis, and multivariate logistic regression analyses were used to test the difference and the correlation between those measures. Time-to-event endpoints (oral feeding) were analyzed by the Kaplan–Meier method.

**Results:**

A total of 75 patients were included in this study. Significant differences were demonstrated in PTD, PRD, SED, STD, HAD, HSD, UESO, PAS, and PRG between supratentorial and infratentorial stroke groups (*p* < 0.05). The PRG score of the lobar intracerebral subgroup was significantly higher (*p* < 0.05) than that of the deep intracerebral and lobar + deep intracerebral stroke subgroups, while HSD was significantly shorter (*p* < 0.01). Spearman’s correlation analysis revealed that PAS was related to PTD, PRG, HAD, and UESO (*p* < 0.05). Multivariate logistic regression analysis demonstrated that HAD and PRG may be risk factors for penetration and aspiration (*p* < 0.05). Kaplan–Meier survival plot showed that there was a significant difference in time to oral feeding between supratentorial and infratentorial stroke groups (*p* < 0.01).

**Conclusion:**

Infratentorial stroke may lead to worse swallowing function as compared with supratentorial stroke, and lobar intracerebral stroke may be worse than deep intracerebral stroke. Suitable preventive measures may be considered for patients with higher PRG scores and shorter HSD to avoid penetration and aspiration.

## Introduction

Stroke is one of the most common causes of death or long-term disability. There may be about 100 million people living with stroke worldwide, which heavily impacts the patients’ quality of life and increases the economic burden (Saini et al., 2021). Post-stroke dysphagia (PSD) occurs in 29–78% of stroke patients and is associated with an increased risk of serious complications, hospital readmissions, and mortality (Cohen et al., 2016; Zhong et al., 2021). Among patients with PSD, aspiration pneumonia, malnutrition, and dehydration are the leading causes of death (Ekberg et al., 2002; Connolly, 2010; Martino et al., 2010).

Different locations of brain lesion are thought to be related to the difference in incidence, severity, and patterns of PSD, in which the brainstem stroke is reportedly the main incentive of PSD (incidence can be up to 40–81%) (Teasell et al., 2002; Zhong et al., 2021). A recent report suggested that lesions in deep brain structure (even a small stroke) were much more frequently accompanied by PSD than lobar cortical lesions (Hess et al., 2021). Another retrospective study also found that infratentorial (including the brainstem and cerebellar) stroke may be more likely to cause serious dysphagia than supratentorial (including deep or lobar intracerebral) stroke (Kim Y. K. et al., 2019). Characteristics of PSD caused by different parts of the brain would be different. These differences can be explained from aspects of mechanism and/or manifestation and captured by multidimensional measures, such as measures for duration, motion, and pharyngeal function from videofluoroscopic swallowing study (VFSS).

Currently, characteristics of PSD in VFSS measures among the brainstem (midbrain, pons, or medulla), cerebellar, deep intracerebral (thalamus or basal ganglia), and lobar (cortex or subcortical areas of the cerebral hemispheres) strokes have not been well described. We, therefore, hypothesized that there were significant differences in VFSS among different stroke lesions through VFSS assessment. By comparison of the multidimensional measures of VFSS, we explored the characteristics of PSD caused by different parts of the brain after stroke. Meanwhile, time-to-oral feeding endpoints were analyzed by the Kaplan–Meier curve to evaluate the difference in prognosis. Furthermore, correlation analysis was used to verify the possible predictive factors of penetration and aspiration after PSD. Altogether we hope to outline some evidence for the forecast of the swallowing function and early intervention of PSD and further provide a reference for how to make the early clinical prevention for serious complications associated with PSD.

## Materials and methods

### Patients

This study received ethical approval from the Third Hospital of Sun Yat-sen University (No. 02-351-01). Data on patients with PSD were from the Department of Rehabilitation Medicine, the Third Hospital of Sun Yat-sen University from November 2018 to March 2021. The possible dysphagia was first screened by well-trained speech therapists specializing in PSD. The diagnostic criteria for PSD were based on parameters in protocols discovered in Silva’s instrument for evaluating dysphagia in stroke patients (Silva, 2004; Clave et al., 2008; Ribeiro et al., 2015). Each evaluator was trained in the VFSS swallowing evaluation procedures. Magnetic resonance imaging (MRI) or computer tomography (CT) was performed to obtain information on infarction, hemorrhage, and lesion sites. Patients were contacted for telephone follow-up until oral feeding with a necessity for the modification of food consistency. According to the lesion sites, the included patients were then divided into the supratentorial group (including lobar, deep intracerebral, and lobar + deep intracerebral stroke subgroups) and infratentorial stroke group (including brainstem, cerebellar, and brainstem + cerebellar stroke subgroups).

### Inclusion criteria and exclusion criteria

Inclusion criteria were as follows: (1) all patients with PSD who underwent at least one VFSS were included in the screening range of this study; (2) age >18 years; (3) patients with stroke due to cerebral hemorrhage and cerebral infarction within 6 months, and the lesion sites after stroke limited to supratentorial or infratentorial stroke; (4) dysphagia was confirmed by VFSS examination and a well-trained speech therapist; and (5) the time interval between CT/MRI and VFSS examinations less than 2 weeks. Exclusion criteria were as follows: (1) CT/MRI suggested brain tumors, neuromyelitis optica, traumatic brain injury, etc.; (2) the lesion sites included both supratentorial and infratentorial intracerebral stroke; (3) no dysphagia was confirmed; (4) the time interval between CT/MRI and VFSS was more than 2 weeks; (5) with structural abnormalities of the oropharynx; (6) with cognitive impairment; and (7) with underlying neurologic disease (such as Parkinson’s disease and dementia).

### Materials

#### Instruments

The VFSS was accomplished by the Lanmage dynamic digital radiography machine (Athena Plus 7500; Shenzhen Lanmage Medical Technology Co., Ltd., Shenzhen, China) for swallowing image acquisition. During VFSS, a metal ball with a diameter of 8 mm was fixed in the neck skin beforehand (at a lateral view equivalent to the C5–6 level) as the reference. Then, the subjects were seated in an upright position, and the lateral images were recorded with fluoroscopy. Radiation exposure ranged up to the top of the nasal cavity, down to the C7 cervical spine, anterior and posterior to the lips and back of the neck. The subjects received 3, 5, and 10 ml of thickened and diluted barium liquid (contrast media: 60% w/v barium sulfate suspension), and the specific steps were performed according to the modified Logemann protocol (Logemann, 1993).

#### Measures

The measures of duration, motion, and pharyngeal function acquired from VFSS were used for the assessment of patients with PSD. Different measures were obtained using ImageJ open-source software (National Institutes of Health, Bethesda, MD, United States). Two investigators worked together on data extraction, if inconsistencies or errors in the records happened, the measures were double-checked and proofread. The same video image was analyzed three times within 2 weeks, and the different measures were the average of three times data extraction. During the analysis, the video was played at a speed of 30 frames/s and played back frame-by-frame; the time and position points were marked for 5 ml of thickened liquid swallowed to obtain the measures of duration, motion, and pharyngeal function.

**FIGURE 1:**
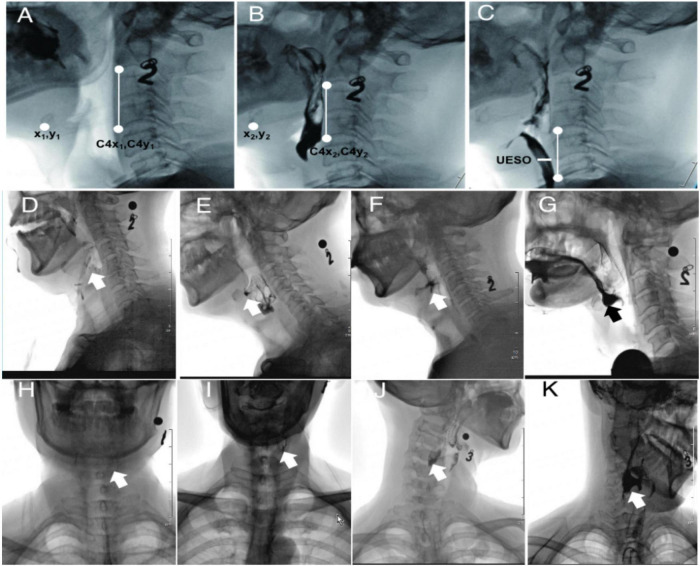
The extraction method of HAD, HSD, UESO, and PRG from VFSS. Number “2” and “3,” oral contrast agent 2 and 3 (thickened liquid); **(A)** hyoid resting position (after rotation); **(B)** highest hyoid position (after rotation); **(C)** the maximum upper esophageal sphincter opening position (after rotation); **(D)** PRG 0 (no residue); **(E)** PRG I (mild residue); **(F)** PRG 2 (moderate residue); **(G)** PRG 3 (severe residue) of the valleculae; **(H)** PRG 0 (no residue); **(I)** PRG 1 (mild residue); **(J)** PRG 2 (moderate residue); and **(K)** PRG 3 (severe residue) of the pyriform sinuses. HAD, hyoid bone anterior-horizontal displacement; HSD, hyoid bone superior-horizontal displacement; UESO, upper esophageal sphincter opening; PRG, Pharyngeal Residual Grade; VFSS, videofluoroscopic swallowing study.

The measures of duration included pharyngeal transit duration (PTD), soft palate elevation duration (SED), pharyngeal response duration (PRD), stage transition duration (STD), and the data extraction according to references (Table 1; Kendall et al., 2000; Suntrup-Krueger et al., 2015; Yagi et al., 2017). The measures of motion included hyoid bone anterior-horizontal displacement (HAD), hyoid bone superior-horizontal displacement (HSD), and upper esophageal sphincter opening (UESO). Due to patient movement and clinician-directed interventions, head movement is typical during VFSS. To account for this variable and avoid erroneous hyoid bone movement estimates, the methods used were as follows: taking the line connecting the anterior-inferior corner of C2 and C4 as the vertical axis, and rotating the line to make it perpendicular to the horizontal axis of the image; marking the positions of the anterior-inferior corner of hyoid bone and C4, respectively—coordinates were (*x*_1_, *y*_1_), (*x*_2_, *y*_2_), (C4*x*_1_, C4*y*_1_), and (C4*x*_2_, C4*y*_2_); and calculating the HAD and HSD by the Equations 1, 2 (see Figures 1A,B), taking the line to connect the anterior-inferior of C4 and C6 as the vertical axis, rotating the image, then marking the width of UESO (see Figure 1C). The metal ball with a diameter of 8 mm was used as a ruler during the analysis.


(1) $$\text{HAD}=\left(x_{2}-x_{1}\right)-\left(\text{C}\,4\,x_{2}-\mathrm{C4}\,x_{1}\right)$$

(2) $$\text{HSD}=\left(y_{2}-y_{1}\right)-\left(\text{C}\,4\,y_{2}-\text{C}\,4\,y_{1}\right)$$

**TABLE 1:** The definition of different measures of duration.

Measures of duration	Definition
Pharyngeal transit duration (PTD)	The bolus head at ramus of mandible to bolus head entering cricopharyngeus
Soft palate elevation duration (SED)	The soft palate migrates forward from its starting position to the end of its backward and downward motion
Pharyngeal response duration (PRD)	The hyoid bone migrates forward from its starting position to the end of its backward and downward motion
Stage transition duration (STD)	The bolus pushes into the ramus of the mandible until maximal excursion of the hyoid begin

The measures of pharyngeal function included Penetration Aspiration Scale (PAS), Pharyngeal Residual Grade (PRG), and Functional Oral Intake Scale (FOIS). PAS is an eight-point rating scale; the higher the score, the more severe the penetration and aspiration symptoms (Seo et al., 2021). The PRG scores were based on a 0–3 scale, in which PRG 0 referred to no residual, PRG 1 referred to <10%, PRG 2 referred to 10–50%, and PRG 3 refers to >50% filling of the valleculae (Figures 1D–G) or pyriform sinuses (Figures 1H–K) by the thickened liquid (Han et al., 2001). FOIS was used to measure the nutritional intake (FOIS score 1 to 3: use of a gastric tube for feeding; 4 to 5: oral feeding with a necessity for the modification of food consistency; and 6 to 7: no modifications in food consistency) (Chen et al., 2019; Souza et al., 2020; Sura et al., 2020). Therefore, in this study, the time-to-event endpoints (oral feeding) refer to FOIS ≥ 4, while FOIS < 4 indicated that a gastric tube was needed.

### Statistical analysis

Continuous variables were tested for normality using the Kolmogorov–Smirnov test. Differences in general characteristics between groups were determined by Pearson’s Chi-square test (χ^2^) or Fisher’s exact test (as appropriate). Collected VFSS data were divided into different subgroups according to the lesion sites as mentioned above and compared with the use of a Kruskal–Wallis (K-W) test (for non-normally distributed data), as appropriate for continuous variables (including PTD, SED, PRD, STD, HAD, HSD, and UESO) or non-continuous variables (including PAS and PRG). The correlation between PAS and the measures of duration, motion, and pharyngeal function was analyzed using the Spearman’s correlation analysis. A strong correlation was defined as >0.50, a moderate correlation was defined as 0.35–0.50, a weak correlation was defined as 0.20–0.34, and a negligible correlation was defined as <0.20 (Davison and Jhangri, 2010). Next, multivariate logistic regression was conducted to identify factors associated with PAS. Furthermore, the time-to-event endpoints (oral feeding) after stroke were assessed using Kaplan–Meier analysis; the patterns of cumulative indwelling gastric tube rates were compared with the use of the log-rank test. Statistical analysis was performed using SPSS software (version 20.0, IBM, New York, NY, United States), and statistical charts were accomplished with the use of GraphPad Prism (version 9.0, GraphPad Software Inc., San Diego, CA, United States). Graphics processing was using ImageJ software version 1.42. *p*-Values below 0.05 were considered to indicate statistical significance.

## Results

**FIGURE 2:**
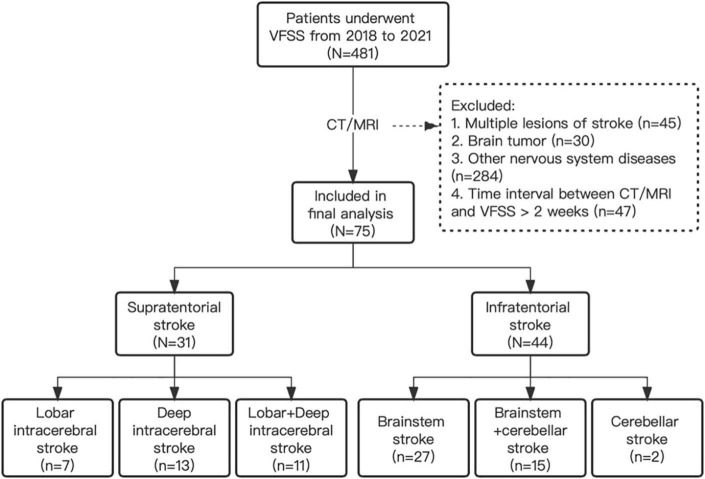
The flow diagram of the study design. VFSS, videofluoroscopic swallowing study.

A total of 75 PSD patients were included in the final analysis, and among them, 31 patients were with supratentorial strokes (7 lobar intracerebral strokes, 13 deep intracerebral strokes, and 11 lobar + deep intracerebral stroke), and 44 patients were with infratentorial strokes (27 brainstem strokes, 2 cerebellar strokes, and 15 brainstem + cerebellar strokes) (Figure 2). In the supratentorial and infratentorial stroke groups, the mean age (±SD) was 66.88 ± 10.64 and 57.34 ± 11.11, respectively (*p* < 0.001, K-W test); the mean (±SD) duration of stroke was 4.60 ± 6.14 and 3.81 ± 4.57 months, respectively (*p* = 0.375, K-W test); and 38.71 and 81.82% patients were diagnosed for ischemic stroke (*p* < 0.001, Chi-square test) (Table 2).

**TABLE 2:** General characteristics of patients.

Variables	Supratentorial (*N* = 31)	Infratentorial (*N* = 44)	*p*
**Age mean** (year) (SD)	66.88 (10.64)	57.34 (11.11)	<0.001
**Gender *n* (%)**			
Male	22 (70.97)	33 (75.00)	0.697
**Stroke type *n* (%)**			<0.001
Ischemic	12 (38.71)	36 (81.82)	
Hemorrhagic	19 (61.29)	8 (18.18)	
**Lesion side *n* (%)**			
Right	12 (38.71)	10 (22.73)	0.326
Left	14 (45.16)	25 (56.82)	
Bilateral	5 (16.13)	9 (20.45)	
**Stroke duration** (month) mean (SD)	4.60 (6.14)	3.81 (4.57)	0.371

### The measures between supratentorial and infratentorial stroke group

**FIGURE 3:**
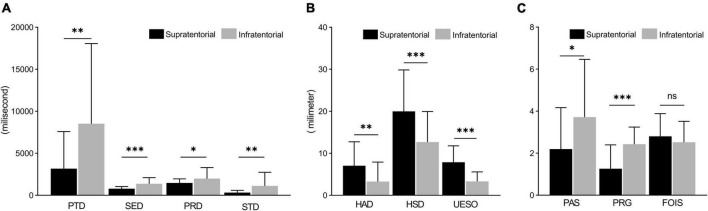
The comparison of measures between supratentorial and infratentorial stroke group. **(A)** Measures of duration (including PTD, SED, PRD, and STD) under a VFSS examination. **(B)** Measures of motion (include HAD, HSD, and UESO) under a VFSS examination. **(C)** Measures of pharyngeal function (including PAS, PRG, and FOIS) under a VFSS examination. PAS, Penetration-Aspiration Scale; FOIS, Functional Oral Intake Scale; PRG, Pharyngeal Residual Grade; PTD, pharyngeal transit duration; HAD, hyoid bone anterior-horizontal displacement; HSD, hyoid bone superior-horizontal displacement; SET, soft palate elevation duration; PRD, pharyngeal response duration; STD, stage transition duration; UESO, upper esophageal sphincter opening. *p < 0.05; **p < 0.01; ***p < 0.001.

For measures of duration, there was a significant difference in PTD (*p* < 0.01), PRD (*p* < 0.01), SED (*p* < 0.001), and STD (*p* < 0.01) between the supratentorial stroke group and infratentorial stroke group (Figure 3A). For measures of motion, a significant difference was found in HAD (*p* < 0.01), HSD (*p* < 0.001), and UESO (*p* < 0.001) between the two groups (Figure 3B). For the measures of pharyngeal function, there were significant differences in PAS (*p* < 0.05) and PRG (*p* < 0.01) scores between the two groups, but no difference in FOIS was found (*p* > 0.05) (Figure 3C).

**FIGURE 4:**
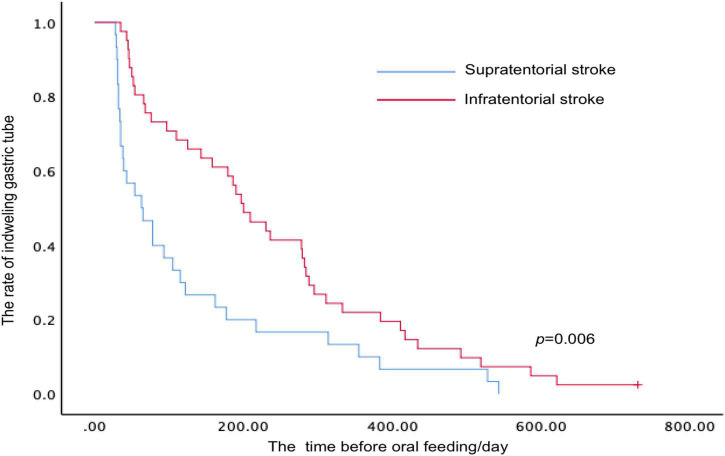
Kaplan–Meier analysis for time-to-event end points (oral feeding with a necessity for the modification of food consistency) stratified by the lesion sites after stroke.

Kaplan–Meier survival analysis was performed to evaluate the time before oral feeding after stroke according to the different lesion sites (including supratentorial and infratentorial stroke) (Figure 4). The mean duration before oral feeding of patients stratified by the lesion sites was 129.63 days (supratentorial stroke group) and 238.62 days (infratentorial stroke group), and a difference was identified between the two groups (*p* = 0.006, log-rank test).

### The measures among lobar, deep intracerebral, and lobar + deep intracerebral stroke subgroups

**FIGURE 5:**
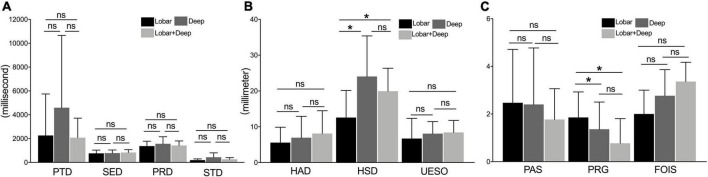
The comparison of measures among lobar, deep intracerebral, and lobar + deep intracerebral stroke subgroup. **(A)** Measures of duration (including PTD, SED, PRD, and STD) under the VFSS examination. **(B)** Measures of motion (including HAD, HSD, and UESO) under the VFSS examination. **(C)** Measures of pharyngeal function (including PAS, PRG, and FOIS) under the VFSS. PAS, Penetration-Aspiration Scale; FOIS, Functional Oral Intake Scale; PRG, Pharyngeal Residual Grade; PTD, pharyngeal transit duration; HAD, hyoid bone anterior-horizontal displacement; HSD, hyoid bone superior-horizontal displacement; SED, soft palate elevation duration; PRD, pharyngeal response duration; STD, stage transition duration; UESO, upper esophageal sphincter opening. *p < 0.05; ns, not significant.

For measures of duration, there was no significant difference among these three subgroups (i.e., lobar, deep intracerebral, and lobar + deep intracerebral subgroups; *p* > 0.05) (Figure 5A). For measures of motion, the HSD of the lobar intracerebral stroke subgroup was significantly shorter than that of the deep intracerebral subgroup and lobar + deep intracerebral stroke subgroup (*p* < 0.05) (Figure 5B). For measures of pharyngeal function, PRG of the lobar intracerebral stroke subgroup was significantly higher than that of the deep intracerebral and lobar + deep intracerebral stroke subgroup (*p* < 0.05) (Figure 5C).

### The measures among the brainstem, cerebellar, and brainstem + cerebellar stroke subgroups

**FIGURE 6:**
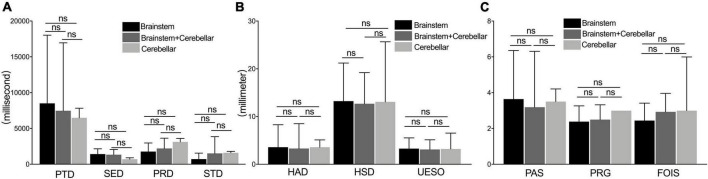
The comparison of measures among the brainstem, cerebellar, and brainstem + cerebellar stroke subgroup. The measures of duration **(A)**, motion **(B)**, and pharyngeal function **(C)** under a videofluoroscopic swallowing study examination between the brainstem stroke group, the brainstem add the cerebellar stroke, and cerebellar stroke subgroup. PAS, Penetration-Aspiration Scale; FOIS, Functional Oral Intake Scale; PRG, Pharyngeal Residual Grade; PTD, pharyngeal transit duration; HAD, hyoid bone anterior-horizontal displacement; HSD, hyoid bone superior-horizontal displacement; SED, soft palate elevation duration; PRD, pharyngeal response duration; STD, stage transition duration; UESO, upper esophageal sphincter opening. ns, not significant.

No significant difference was found in the measures of duration, motion, and pharyngeal function among brainstem, cerebellar, and brainstem + cerebellar stroke subgroups (*p* > 0.05) (Figures 6A–C).

### Risk factors related to penetration and aspiration after stroke

**FIGURE 7:**
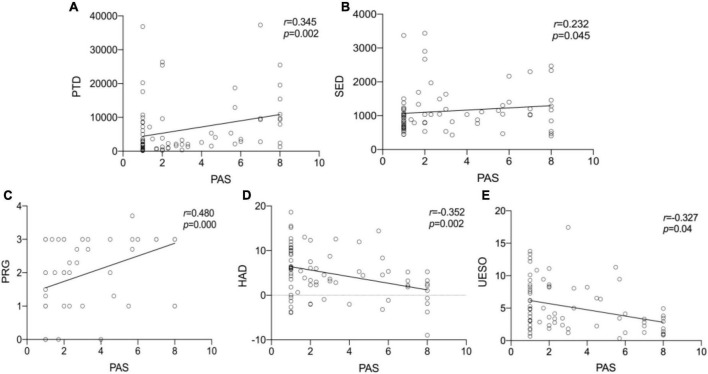
The Spearman correlation analysis for PTD, SED, PRG, HAD, UESO, and PAS. The correlation between PTD and PAS **(A)**. The correlation between SED and PAS **(B)**. The correlation between PRG and PAS **(C)**. The correlation between HAD and PAS **(D)**. The correlation between UESO and PAS **(E)**. There was a significant correlation between PAS and PTD, PRG, HAD, UESO, among which PRO exhibited moderate correlation. PAS, Penetration-Aspiration Scale; PRG, Pharyngeal Residual Grade; PTD, pharyngeal transit duration; HAD, hyoid bone anterior-horizontal displacement; SED, soft palate elevation duration; UESO, upper esophageal sphincter opening.

We observed statistically significant relationships between PAS scores and PRG (Spearman’s correlation coefficient, *r* = 0.480, *p* < 0.001), PTD (*r* = 0.345, *p* = 0.002), SED (*r* = 0.232, *p* = 0.045), HAD (*r* = −0.352, *p* = 0.002), and UESO (*r* = −0.327, *p* = 0.04). There was only a moderate correlation between change in PAS and change in PRG (*r* = 0.480, *p* < 0.001), and HAD (*r* = −0.352, *p* = 0.002) (Figure 7).

Interestingly, in the logistic regression model, we further found a significant difference between PAS and PRG (*p* = 0.019), and PAS and HAD (*p* = 0.046), suggesting PRG and HAD were likely to be more closely related to outcomes. In addition, no significant difference between PAS and other measures was found (Table 3).

**TABLE 3:** The logistic regression analysis between different measures and PAS.

Variables	Regression coefficient	95% CI	*p*
PTD	0.000028	(−0.000030, 0.000086)	0.344
SED	−0.000090	(−0.000813, 0.000632)	0.807
PRG	0.654778	(0.106584, 1.2029730)	0.019
HAD	−0.091324	(−0.181052, −0.001596)	0.046
UESO	0.004550	(−0.153089, 0.162189)	0.955

Test of parallelism, p = 0.277. PTD, pharyngeal transit duration; HAD, hyoid bone anterior-horizontal displacement; SED, soft palate elevation duration; UESO, upper esophageal sphincter opening; PAS, Penetration-Aspiration Scale; PRG, Pharyngeal Residual Grade; 95% CI, 95% confidence interval.

## Discussion

The main findings of this retrospective study were given as follows: (i) infratentorial stroke may lead to worse swallowing function as compared with supratentorial stroke, which mainly manifested as longer duration of swallowing (including longer PTD, PRD, SED, and STD), shorter motion displacement (including shorter HAD, HSD, and UESO), higher swallowing scores (including higher PAS and PRG), and longer time before oral feeding; (ii) swallowing dysfunction caused by lobar intracerebral stroke may be worse than deep intracerebral stroke; and (iii) higher PRG and shorter HAD may be the risk factors for penetration and aspiration.

Infratentorial stroke may lead to worse swallowing function as compared with supratentorial stroke. Infratentorial stroke usually refers to a stroke occurring below the tentorium cerebelli (including the brainstem and cerebellar) (Kim H. Y. et al., 2019). According to previous studies, the medulla oblongata is believed to be the swallowing center pattern generator (swCPG), in which the nucleus tractus solitarius (NTS) is a sensory nucleus responsible for swallowing initiation, while the nucleus ambiguus (NA) is a motor nucleus. Both NTS and NA participate in the coordinated contraction of lingual, pharyngeal, and laryngeal muscles (Jean, 2001; Zhang M. et al., 2021). In general, the brain regions related to swallowing include the cerebral cortex, cerebellum, pons, medulla oblongata, and so on. Among them, the cerebellum plays a regulatory function through the following two neural pathways (Flowers et al., 2017). First, the cerebral cortex transmits signals to the pontine nucleus on the contralateral cerebellar hemisphere, following to the middle cerebellar peduncle, while the cerebellar Purkinje fibers transmit the signals to the deep dentate nucleus, then conduct them upward to the dorsolateral thalamus, and finally transmit from the thalamus to the contralateral cerebral cortex. Second, the cerebellum may also regulate swallowing through cerebellum-inferior olive-swCPG neural pathway (Rahmati et al., 2018). Any damage to these neural pathways may cause the decrease of inhibitory effect by the cerebellum on the cerebral cortex, leading to dysphagia (Roostaei et al., 2014). This study found that infratentorial stroke exhibited longer duration of swallowing (including longer PTD, PRD, SED, and STD), shorter motion displacement (including shorter HAD, HSD, and UESO), higher swallowing scores (including higher PAS and PRG), and longer time before oral feeding, indicating infratentorial stroke may be more serious compared with the supratentorial stroke. However, no significant difference was found among the brainstem, cerebellar, and the brainstem + cerebellar stroke subgroups, probably because brainstem stroke would directly lead to the impairment of swCPG, while cerebellar stroke indirectly affects the swCPG (Rahmati et al., 2018). Both the brainstem and cerebellar are vital for the maintenance of normal swallowing function. In short, this finding draws attention to the need for early functional assessment of swallowing to avoid serious complications like aspiration pneumonia, especially for patients with infratentorial stroke.

For the supratentorial stroke group, we found that swallowing dysfunction caused by lobar intracerebral stroke may be worse than deep intracerebral stroke. In clinical practice, however, some patients who exhibit intact brainstem function still suffered from PSD (Wilmskoetter et al., 2019). The lobar intracerebral and deep intracerebral neural networks were reportedly related to PSD (including sensorimotor cortex, insular, prefrontal lobe, thalamus, supplementary motor area, and superior temporal gyrus) (Dehaghani et al., 2016; Galovic et al., 2017; Lee et al., 2020). Functional magnetic resonance imaging (fMRI) also suggested that the activation of the primary sensory/motor cortex may predominate in reflex swallowing, while the extensive activation of areas including primary sensory/motor cortex, insular, prefrontal lobe, subgenual cingulate gyrus, cuneate, and precuneus may predominate in spontaneous swallowing (Dionisio et al., 2019; Hess et al., 2021). This study found that the HSD of the lobar intracerebral stroke subgroup was significantly shorter than that of the deep intracerebral stroke subgroup, indicating that the sensorimotor cortex and other cortex lesions mentioned above could have led to worse swallowing function compared with deep intracerebral stroke, but the mechanism has not yet been fully understood. A possible reason is that the lobar intracerebral participated in the afferent sensory and the motor efferent signals process, which may result in a shorter HSD (Kiernan, 2012; Wilmskoetter et al., 2019). Furthermore, this study also found that simple lobar intracerebral stroke patients exhibit a higher PRG score compared with the deep intracerebral stroke group. Previous research found that the parietal lobe and the temporal lobe may be associated with pharyngeal remnants, while the connections and integration among multiple brain regions are involved in the coordination of swallowing function, which may cause the higher PRG scores in the lobar intracerebral stroke group (Suntrup-Krueger et al., 2017; Wilmskoetter et al., 2019).

Higher PRG and shorter HAD may be the risk factors for penetration and aspiration. We used Spearman’s correlation analysis to assess the correlation between PAS and different measures of VFSS and found that there was a statistical relevance between PAS and PTD, PRG, HAD, and UESO, in which there was a moderate correlation between PAS and PRG, HAD. Logistic regression analysis further suggested that PRG and HAD may be the risk factors for penetration and aspiration. This appears to be reasonable, because patients with increased pharyngeal residue usually required repeated swallows to clear the pharyngeal residue thickened liquid during VFSS examination, which may increase the risk of penetration and aspiration. Second, patients with more pharyngeal residue are more likely to experience aspiration throughout the rehabilitation process even if no aspiration happens during the VFSS examination. Meanwhile, the aging individuals often exist a shorter hyoid bone displacement (Kim and McCullough, 2008; Kang et al., 2010; Molfenter and Steele, 2011), which may serve to significantly aggravate aspiration and lengthen the rehabilitation process. Therefore, the shorter HAD might be the risk factor for penetration and aspiration, which supported the validity of our original work and was consistent with the study from Zhang Z. et al. (2021). However, more research is needed to find the exact relationship between hyoid bone displacement and aspiration risk (Zhang et al., 2020).

Suitable preventive and treatment measures may be considered for patients with PSD. First, as infratentorial stroke may lead to worse swallowing function, a comprehensive assessment is therefore needed before oral feeding to avoid serious complications. Second, foods with a certain viscosity are recommended due to a higher risk of aspiration in patients with higher PRG and HAD (McCarty and Chao, 2021). Third, the shorter HSD and HAD mean a decrease in the laryngeal activity, which can be relieved by treatment methods, such as a change in the size of food bolus, supraglottic swallow, super-supraglottic swallow, and surface electromyography biofeedback (Logemann, 1993). Forth, for the decrease of UESO, a change in the size and viscosity of food bolus is recommended, and balloon dilatation and botulinum toxin injection are also reportedly useful for patients. These countermeasures may be beneficial for patients with PSD (Lan et al., 2013; Xie et al., 2022).

### Limitations

There are some limitations of this retrospective study. First, the time from stroke onset to VFSS was not strictly limited, which may lead to bias in the results. Second, the different volume of thickened liquids was adopted during the VFSS examination, but only the measures from 5 ml thickened liquid were included in the final analysis to avoid bias. Third, the detailed information (including lesion size and specific lesion) about lobar and deep intracerebral lesions after stroke was not well described in this study due to the limited data. Fourth, the evaluation of swallowing function in the study is limited to VFSS, instead of the comprehensive evaluation, such as fiber optic endoscopic evaluation and high-resolution manometry. Therefore, more high-quality prospective studies are urgently needed.

## Conclusion

Infratentorial stroke may lead to worse swallowing function as compared with supratentorial stroke, and lobar intracerebral stroke may be worse than deep intracerebral stroke. More attention should be paid to patients with infratentorial stroke in clinical practice, and suitable preventive measures may be considered for patients with higher PRG scores and shorter HSD to avoid penetration and aspiration.

## Data availability statement

The raw data supporting the conclusions of this article will be made available by the authors, without undue reservation.

## Ethics statement

The studies involving human participants were reviewed and approved by The Third Hospital of Sun Yat-sen University. The ethics committee waived the requirement of written informed consent for participation. Written informed consent was obtained from the individual(s) for the publication of any potentially identifiable images or data included in this article.

## Author contributions

JQ was responsible for article retrieval and writing. Z-MW was responsible for his professional assistance in language editing, statistical analysis, and illustration drawing during revision. Q-PY, MD, YD, and Z-TH were responsible for the selection of articles and analysis of the data. Z-LD was responsible for the review of articles and ensuring that all authors have approved the manuscript before submission. All authors contributed to the article and approved the submitted version.

## Abbreviations

PSD: post-stroke dysphagia
VFSS: videofluoroscopic swallowing study
PAS: Penetration Aspiration Scale
FOIS: Functional Oral Intake Scale
PRG: Pharyngeal Residual Grade
HAD: hyoid bone anterior-horizontal displacement
HSD: hyoid bone superior-horizontal displacement
PTD: pharyngeal transit duration
SED: soft palate elevation duration
PRD: pharyngeal response duration
STD: stage transition duration
UESO: upper esophageal sphincter opening
NTS: nucleus tractus solitarius
swCPG: swallowing center pattern generator
NA: nucleus ambiguus.
